# Immunotherapeutic Strategies in Chronic Lymphocytic Leukemia: Advances and Challenges

**DOI:** 10.3389/fonc.2022.837531

**Published:** 2022-02-21

**Authors:** Francesca Perutelli, Rebecca Jones, Valentina Griggio, Candida Vitale, Marta Coscia

**Affiliations:** ^1^ University Division of Hematology, Azienda Ospedaliera Universitaria (A.O.U.) Città della Salute e della Scienza di Torino, Torino, Italy; ^2^ Department of Molecular Biotechnology and Health Sciences, University of Torino, Torino, Italy

**Keywords:** chronic lymphocytic leukemia, immunotherapy, monoclonal antibodies, CAR T cells, CAR NK cells

## Abstract

Immune-based therapeutic strategies have drastically changed the landscape of hematological disorders, as they have introduced the concept of boosting immune responses against tumor cells. Anti-CD20 monoclonal antibodies have been the first form of immunotherapy successfully applied in the treatment of CLL, in the context of chemoimmunotherapy regimens. Since then, several immunotherapeutic approaches have been studied in CLL settings, with the aim of exploiting or eliciting anti-tumor immune responses against leukemia cells. Unfortunately, despite initial promising data, results from pilot clinical studies have not shown optimal results in terms of disease control - especially when immunotherapy was used individually - largely due to CLL-related immune dysfunctions hampering the achievement of effective anti-tumor responses. The growing understanding of the complex interactions between immune cells and the tumor cells has paved the way for the development of new combined approaches that rely on the synergism between novel agents and immunotherapy. In this review, we provide an overview of the most successful and promising immunotherapeutic modalities in CLL, including both antibody-based therapy (i.e. monoclonal antibodies, bispecific antibodies, bi- or tri- specific killer engagers) and adoptive cellular therapy (i.e. CAR T cells and NK cells). We also provide examples of successful new combination strategies and some insights on future perspectives.

## 1 Introduction

Chronic Lymphocytic leukemia (CLL) is the most common hematological disorder in the Western world, with an incidence of 4.2/100 000/year that increases to more than 30/100 000/year at an age of >80 years (1). Treatment options for CLL patients have enriched and developed over the time, starting from the standard chemotherapy-based approaches containing alkylating agents (2) and purine analogues (3). Despite its potent anti-tumor activity, traditional chemotherapy alone has not shown any improvement in the overall survival (OS) of CLL patients (3). The addition of the anti-CD20 monoclonal antibody (mAb) rituximab to chemotherapy has determined significant advances in terms of overall response (OR) and OS rates (4), demonstrating for the first time the therapeutic efficacy of strategies exploiting the immune system as a weapon to eliminate tumor cells.

Herein, we will go through a summary of immune-based approaches explored over the time for the treatment of CLL, also discussing the most recent, successful and/or promising advances made in the field of immunotherapy.

## 2 Overview of Immunotherapeutic Approaches in CLL

**Figure 1:**
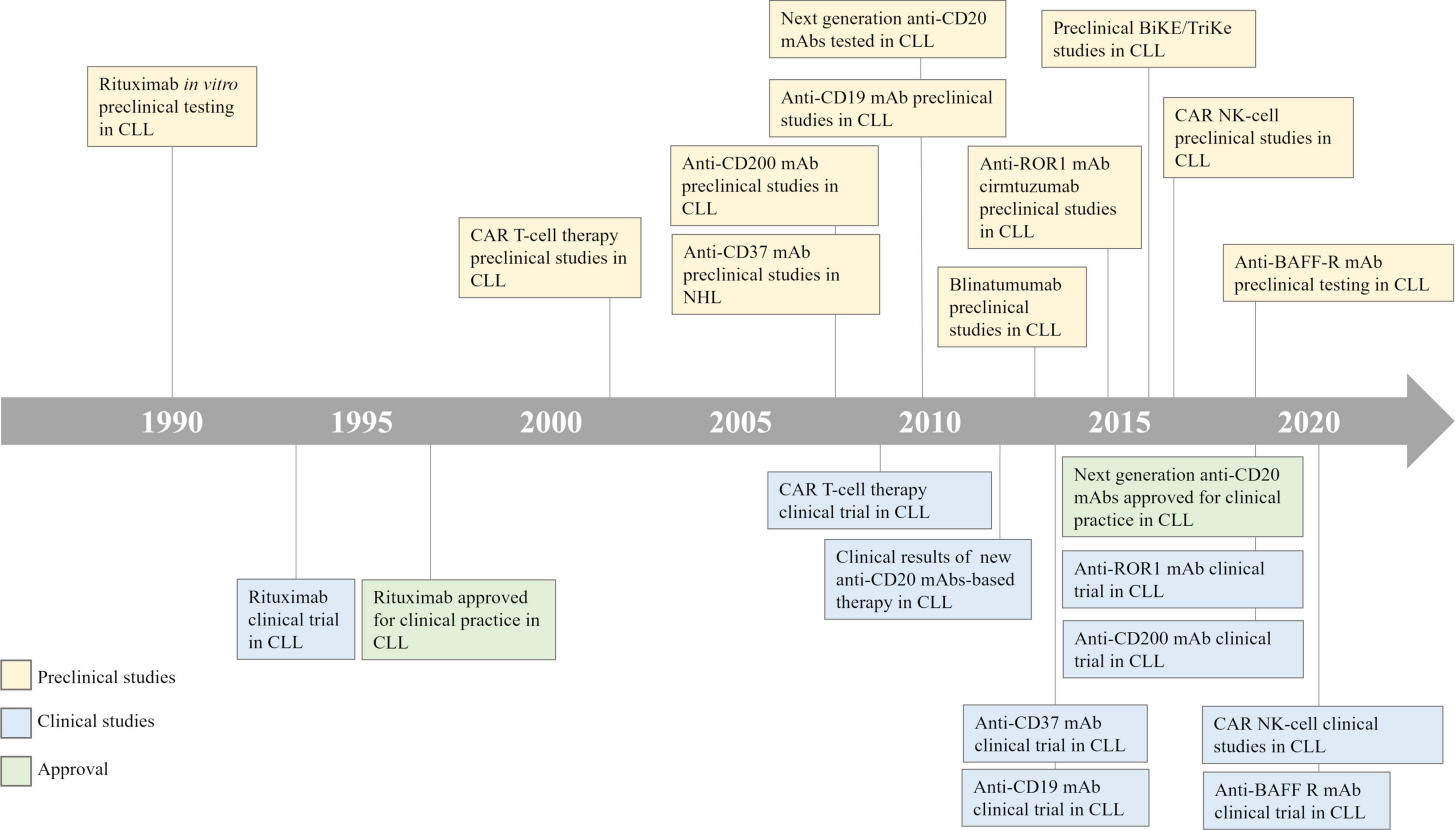
CLL treatment timeline. A timeline illustrates the development of immune-based therapeutic strategies for the treatment of patients with CLL. CLL, Chronic Lymphocytic Leukemia; CAR, Chimeric Antigen Receptor; NHL, Non-Hodgkin Lymphoma; BiKE/TriKE, Bispecific/Trispecific Specific Killer Engagers; NK, Natural Killer.

The concept of boosting immune responses against hematologic tumors is more than 50 years old, when E. Donnall Thomas performed the first allogeneic hematopoietic stem cell transplantation (HSCT) in 1957 (5). The efficacy of HSCT is mediated by the graft *versus* leukemia (GvL) effect driven by the donor transferred T cells, which are able to recognize and eliminate leukemia cells (6). In CLL, the GvL efficacy was established by several evidences, as the lower risk of relapse observed in patients who develop chronic graft *versus* host disease (GvHD) (7), and the possibility to potentiate the GvL effect through donor lymphocyte infusion (8). Nowadays, thanks to the availability of several successful therapeutic options, HSCT is reserved only to young and fit CLL patients with high-risk disease features [e.g. del(17p) and/or *TP53* mutations] and showing progression or resistance to both chemotherapy- and novel agents-based prior treatment regimens (9). Given the evidence of an immune-mediated effect of HSCT, several efforts have been made to develop new strategies that exploit or elicit anti-tumor immune responses against hematologic disorders, as CLL. In **Figure 1** we have provided a timeline of the clinical and preclinical studies in CLL.

Tumor-specific active immunotherapy, which consists in the administration of tumor-derived component/s or of whole tumor cells in the form of an anti-tumor vaccine, with the aim of actively boosting the host’s immunity against the tumor cells, has been investigated in hematological malignancies, including CLL. Despite encouraging preliminary results obtained (10, 11), both tumor cell- and dendritic cell-based vaccine formulations failed to produce reproducible clinical effects (10), possibly due to the presence of meaningful alterations linked to tumor escape mechanisms in the immune system of CLL patients.

Non-specific active immunotherapy, which is the administration of agents with broad immunomodulatory properties - such as cytokines and chemokines - with the aim of overcoming tumor escape mechanisms and producing an immune attack against malignant cells, had been explored in hematologic malignancies (12). Immune checkpoint inhibitors (ICI) can be considered as a more recent form of non-specific active immunotherapy, due to their ability to elicit anti-tumor responses by blocking inhibitory receptors on immune cells or their ligands on cancer cells. In the context of hematological disorders, ICI have brought significant benefits in multiple settings, especially in Hodgkin lymphomas and diffuse large B-cell lymphomas (DLBCL) (13, 14). However, in CLL, even if preclinical data had demonstrated an anti-tumor effect exerted by antibodies targeting PD-L1 or PD1 and LAG3 (15, 16), clinical trials have produced disappointing results (17, 18), and today ICI are rarely considered an option for patients with CLL.

Similarly, immunomodulatory drugs (IMIDs) can also be classified as a potential non-specific active immunotherapy. IMIDs have shown to induce pleiotropic effects on the immune system of CLL patients (19), such as down-regulation of tumor cell–inhibitory molecules, recovery of the impaired T-cell functions and normalization of the number of different T-cell subsets in lenalidomide-treated patients (20, 21). Avadomide, a next-generation IMID, was reported to trigger anti-tumor T cell-mediated immune responses when combined with checkpoint inhibitors in CLL preclinical models (22–24). These findings suggest that, despite the low direct anti-tumor activity, IMIDs might still represent a promising option for CLL treatment when used in the context of combination regimens exploiting their immune-mediated anti-tumor functions.

In the context of passive immunotherapy, which consists in the administration of mAbs that selectively target antigens broadly expressed on the surface of tumor cells, rituximab was the first anti-CD20 mAb approved for clinical use - followed by the newer ofatumumab, obinutuzumab and ublituximab. To date, studies are searching for novel strategies aimed at more effectively directing the anti-tumor power of effector cells, such as T cells or NK cells, through the targeting of B-cell specific antigens. In this context, innovative approaches currently under development are i) bi- and tri-specific T- and NK-cell engagers, and chimeric antigen receptor (CAR)-modified T and NK cells. In this review we will provide an overview of these immunotherapeutic modalities in CLL, their current role in patients management, and future perspectives.

## 3 Successful Immunotherapy Options in CLL

Thanks to their successful results, antibody-based therapies and adoptive cellular therapies currently represent the main field of investigation in the context of immunotherapy in CLL.

**Figure 2:**
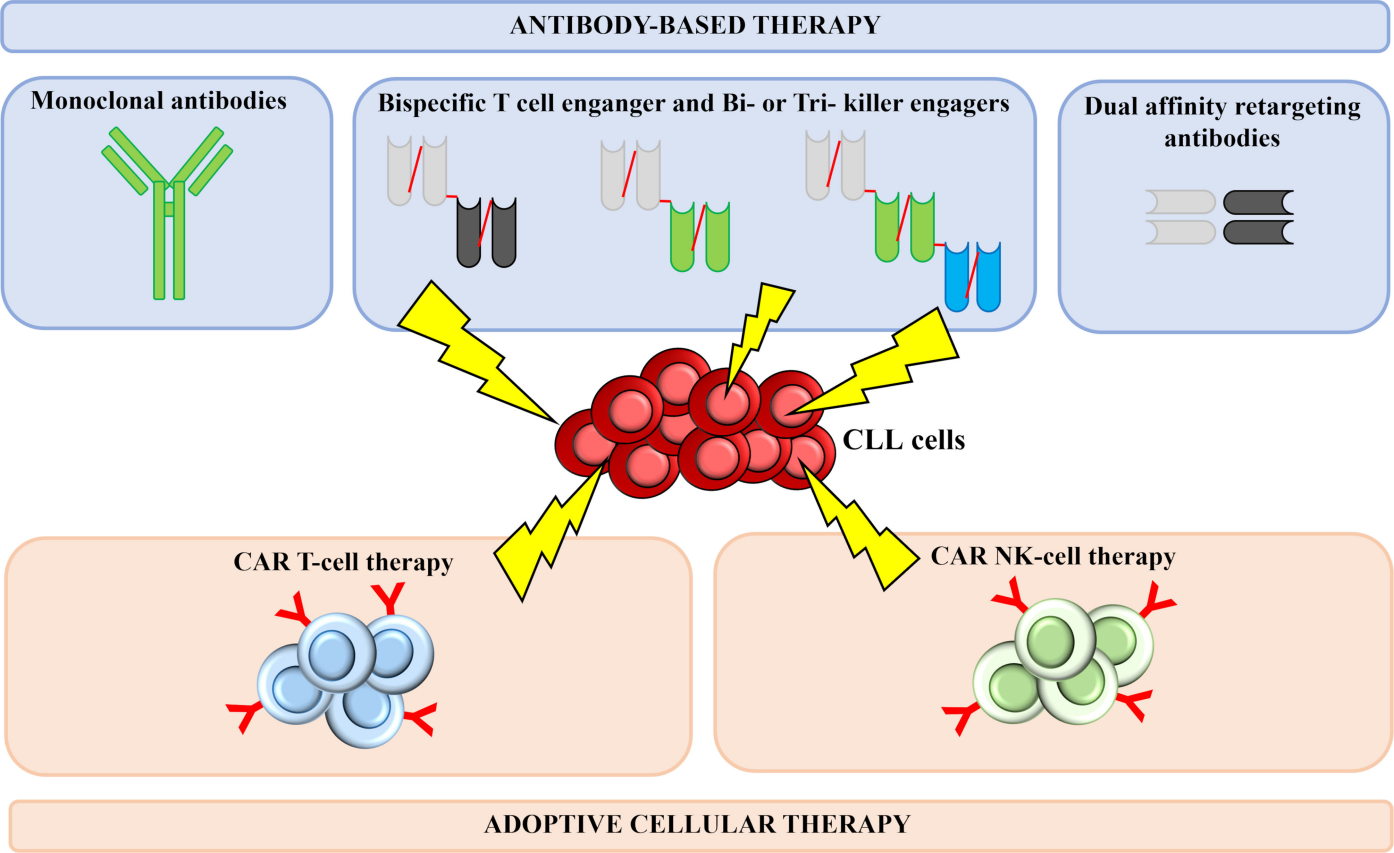
Successful immunotherapeutic strategies implemented in CLL. Several immune-based therapeutic strategies are under development for CLL treatment. Thanks to their successful results, antibody-based therapies and adoptive cellular therapies currently represent the main field of investigation in the context of CLL. Monoclonal antibodies act by binding a specific antigen expressed on the surface of leukemia cells, thus generating cytotoxic responses. Bispecific T cell-engagers are small antibody-based molecules that contain two antigen-binding domains capable of redirecting T cells against antigen-bearing cancer cells. Bi- and tri-specific killer cell engagers consist of two or three antigen-recognition domains and are capable to simultaneously target a tumor cell antigen and a molecule expressed on the surface of NK cells, with the aim of triggering immune cells against tumor cells. DART are designed in a criss-cross format in order to improve pharmacokinetic profile and T-cell killing. CAR T and CAR NK cells are T lymphocytes and NK cells engineered to express a chimeric receptor, able to recognize a tumor surface antigen; upon antigen recognition and activation of the costimulatory domains, a cytotoxic response is activated, leading to leukemia cells killing. CLL, chronic Lymphocytic Leukemia; CAR, Chimeric Antigen Receptor; NK, Natural Killer.

Herein, we analyze in more detail the most promising immunotherapeutic approaches currently under development (**Figure 2**), including mAbs, bispecific antibodies (bsAbs), bi- or tri- specific killer engagers (BiKEs and TriKEs), and CAR T and CAR NK cells.

### 3.1 Antibody-Based Therapy

#### 3.1.1 Monoclonal Antibodies: First Successful Example of Passive Immunity

MAbs are the first immunotherapeutic weapon which gave successful results in CLL. The anti-tumor effect of mAbs relies on several mechanisms, including antibody-dependent cellular cytotoxicity (ADCC), antibody-dependent cellular phagocytosis, complement-dependent cytotoxicity or direct pro-apoptotic effects (25). The selection of the best candidate antigens, together with the technical advancements aimed at improving mAbs formats, are two key steps required for the development of successful mAb-based therapies.

CD20 is a molecule expressed on B cells’ surface, but not on precursor B-cells or plasma cells, and it is the first target antigen explored in the setting of CLL (26). The combination of the anti-CD20 mAb rituximab with chemotherapeutic agents had induced long-term remissions and highly relevant improvement in OS in specific subgroups of CLL patients (27), and to date, despite the advent of novel agents, the combination FCR is still often considered the standard therapeutical method for previously untreated, fit patients with a low-risk disease (4, 28). Aimed at improving the efficacy of rituximab, next generation CD20-targeting mAbs have been developed. Ofatumumab, obinutuzumab and ublituximab have shown efficacy in phase 2 and/or phase 3 clinical trials when used in combination with conventional chemotherapy (29–31), Bruton tyrosine kinase (BTK) inhibitors (32–35), PI3K inhibitors (36, 37) or venetoclax (38). Despite their promising results in clinical practice, the use of anti-CD20 mAbs might be limited by several factors, including (i) the presence of high levels of circulating soluble CD20 antigen, that may interfere with the binding of the mAb to leukemic cells (39), (ii) the selection of antigen loss variants in rituximab-treated patients (40), and (iii) the presence of defective complement components, which affects mAb-induced cytotoxicity (41).

In order to broaden the number of mAb-targeted antigens, new perspectives are being brought in the setting of the antibody-based therapies in CLL.

When studying another commonly targeted antigen as CD19, we are able to distinguish it from CD20 as CD19 is a pan-B surface antigen expressed also on precursor B cells. Even if CD19 is considered as a promising antigen to be targeted in the context of CAR T-cell therapy, CD19-directed mAbs did not show competent cytotoxic effects, possibly due to the rapid internalization of CD19 (42). Nevertheless, currently two different Fc-engineered anti-CD19 mAbs are under investigation in clinical studies. Inebilizumab is an affinity-optimized anti-CD19 mAb that showed promising results in previously treated CLL patients in a phase 1 study (43). However, phase 2 trials testing inebilizumab in combination with chemotherapy did not find any significant differences compared to rituximab plus chemotherapy regimens (25). A different anti-CD19 mAb is tafasitamab, which has an engineered Fc region that enhances CD16 binding affinity. A phase 2 trial evaluating the combination of tafasitamab with lenalidomide is currently ongoing (NCT02005289), and preliminary data of the phase 2 trial testing tafasitamab combined with ibrutinib or venetoclax in CLL patients refractory to BTK inhibitors reported promising results in terms of ORR (44).

An alternative target currently under investigation is CD37, a molecule that, similarly to CD20, is expressed on the surface of mature B cells. Otlertuzumab is an anti-CD37 fusion protein derived from the chimeric protein SMIP-016, and it is characterized by the full binding activity of a mAb at a one-third of the regular antibody size (45). Otlertuzumab appears to be well tolerated in a phase 1 trial in both treatment-naïve and pre-treated CLL patients (46); furthermore, otlertuzumab combined with bendamustine showed a significant efficacy in a phase 2 trial in patients with relapsed or refractory CLL (47). BI 836826 is another CD37-targeting mAb that has been Fc-engineered in order to improve its cytotoxic effects. BI 836826 has proven to be particularly effective in CLL patients with del(17p) and/or *TP53* mutation in a phase 1 study, and more recently a clinical trial testing its combination with ibrutinib in relapsed or refractory CLL patients has been terminated (48). Last, the Fc-engineered DuoHexaBody-CD37 is a biparatopic (dual epitope-targeting) anti-CD37 mAb with the E430G mutation that exerts enhanced cytotoxic functions (49). DuoHexaBody-CD37 displayed potent anti-tumor activity *in vivo* in both cell line- and patient-derived xenograft CLL models (49) and a first-in-human clinical trial is currently ongoing (NCT04358458).

A particularly appealing target for immunotherapy of CLL is the surface molecule CD200, which is not only broadly expressed on leukemic cells (50), but it is also an immunoregulatory receptor dampening immune responses and contributing to maintaining self-tolerance. Results from a phase 1 study demonstrated that treatment with samalizumab, a CD200-directed mAb, was associated with a good safety profile and reduction of tumor burden in the majority of patients with advanced CLL (51).

A recent target being explored in patients with CLL is the receptor tyrosine kinase-like orphan receptor 1 (ROR1), that is selectively expressed only on cancer cells. The combination of ibrutinib with cirmtuzumab, a ROR1-directed mAb, was investigated in a phase 1/2 trial, showing a good tolerability together with a significant efficacy (52, 53). Interestingly, the anti-ROR1 antibody-drug conjugate zilovertamab vedotin (VLS-101) has shown a strong activity in xenograft models of CLL transformed into Richter syndrome (RS) (54), thus providing the basis for the currently ongoing phase 1 clinical trial investigating VLS-101 safety and efficacy in patients with hematologic malignancies, including CLL (NCT03833180).

B cell–activating factor (BAFF) is an immunomodulatory cytokine involved in the regulation of B-cell signal and activation. Recently, it has been shown that BAFF can mediate resistance of CLL cells to new targeted agents by sustaining survival and anti-apoptotic signals of leukemic cells (55). The anti-BAFF mAb belimumab, which is approved for treatment of systemic lupus erythematosus, when combined with idelalisib, ibrutinib and venetoclax for the treatment of patients with CLL, has shown to increase the sensitivity of the malignant cells to all three targeted agents (56). The importance of the BAFF/BAFF-receptor (BAFF-R) axis has been shown also by studies evaluating the efficacy of molecules targeting the BAFF-R. BAFF-R might be an ideal candidate because it is expressed on B cells, but not on their precursors (57). In preclinical studies, ianalumab, an anti-BAFF-R mAb, showed superior activity compared to CD20- and CD52-directed mAbs, and its combination with ibrutinib produced prolonged survival compared with either therapy alone in preclinical models (58). Today, the regimen combining ianalumab with ibrutinib is under evaluation in a phase 1 study (NCT03400176). Preliminary results from this trial show an acceptable safety profile and a good activity of the combination, and provide evidence of the possibility of discontinuing ibrutinib by adding ianalumab, thus leading to a fixed-duration ibrutinib-based regimen (59). Further ongoing investigations will provide more information on the combination of ibrutinib and ianalumab, also for the treatment of previously untreated patients with CLL.

#### 3.1.2 Bispecific Antibodies: A Bridge Between Passive and Active Immunity

A new promising class of antibody-based therapy is bsAbs, molecules that combine antibody directed therapies with cellular mediated immunotherapy. A bsAb consists of two variable regions, in which one binds effector cells and the other a tumor associated antigen, resulting in a new immunological synapse aimed at inducing tumor cell lysis. BsAbs include bispecific T-cell engagers (BiTEs) and dual affinity retargeting antibodies (DARTs), which present a more favorable pharmacokinetic profile when compared to BiTEs.

It has been broadly described that the T-cell compartment of CLL patients is affected by several immune defects, including an impaired immunologic synapse formation, expression of exhaustion markers as well as overexpression of inhibitory immune checkpoints (60). Therefore, immunotherapies that trigger an empowered T-cell-mediated response may represent a valid strategy for the treatment of CLL.

The first bsAb tested in CLL was blinatumomab, a CD19/CD3 bsAb designed with the BiTE format. Preclinical studies revealed that blinatumomab possesses a potent anti-tumor activity, being able to effectively eliminate CLL cells in a mouse-xenograft model (61) and in samples from both treatment-naïve and previously treated CLL patients (62). From the clinical standpoint, blinatumomab demonstrated to be effective in a case of refractory RS as a bridge to HSCT (63) and to be safe and well tolerated in a phase 1 clinical trial enrolling patients with relapsed or refractory B-cell non-Hodgkin lymphomas (NHL) (64). Specific data on the tolerability and efficacy of blinatumomab in CLL patients are currently not available, although two ongoing clinical trials are evaluating the use of blinatumomab in combination with lenalidomide (NCT02568553) or of blinatumomab expanded T cells (NCT03823365) in patients with a broad spectrum of NHL, including CLL.

Recently, another bsAb, the MGD011 CD3xCD19 DART (also known as JNJ-64052781) displayed a good *in vitro* efficacy in terms of CLL cells killing by engaging CLL-derived T cells to fight the tumor (65). These preclinical results indicated that MGD011 was capable to partially restore immunological dysfunctions of T cells from CLL patients, resulting in the induction of their activation and proliferation markers. Additionally, MGD011 induced a non-apoptotic killing of leukemic cells, thus showing efficacy also in eliminating CLL cells resistant to venetoclax (65).

Interestingly, the anti-leukemic activity of bsAbs has shown to be effectively potentiated by BTK inhibitors. In particular, T cells isolated from patients receiving ibrutinib for longer than 6 months and co-cultured with autologous CLL cells and anti-ROR1 BiTE demonstrated an enhanced cytotoxicity compared to T cells from non-ibrutinib treated patients (66). Similarly, recent data have shown that both ibrutinib and acalabrutinib are able to potentiate the activity of CD19/CD3 bsAb in CLL models (67). These observations suggest that reversal of baseline dysfunctions of autologous patient-derived T cells is required to gain a full anti-tumor activity of BiTEs.

A novel promising approach consists in the administration of a bispecific antibody targeting leukemic cells and Vγ9Vδ2 T-cells, a conserved T-cell subset with potent intrinsic anti-tumor properties. In the preclinical setting, a CD40 bispecific γδ T-cell engager has shown to induce a powerful Vγ9Vδ2 T cell-dependent anti-leukemic response, also preventing CD40/CD40L-induced pro-survival signaling (68). More recently, a CD1d-specific Vγ9Vδ2-T cell engager based on single-domain antibodies has been explored in preclinical CLL models, showing its capability of determining cytokine production and degranulation by Vγ9Vδ2-T cells from both CLL patients and controls, and of inducing CD1d-dependent tumor lysis (69). Consistently with previous studies (70), the addition of ATRA increased CD1d expression and potentiated Vγ9Vδ2 T-cell engager-induced cytotoxicity of CLL cells (69).

Taken together, these results show that bsAbs may potentially represent a valid option for the treatment of CLL patients, and particularly for the cohort of high-risk patients with a poor prognosis and who have acquired resistance to previous therapies.

#### 3.1.3 Bi- or Tri-Specific Killer Engagers: Directing Innate Immunity Against CLL Cells

In CLL patients, NK cells are reported to be hypofunctional, with impairments in target cells recognition, direct cellular cytotoxicity and cytokine production (60). Indeed, improving NK-cell cytotoxic functions represents a good immunotherapeutic option. Similar to bsAbs, BiKEs and TriKEs can recruit NK cells to target tumor antigens.

TriKEs targeting the NKG2D receptor ligand ULBP2 (ULBP2/aCD19/aCD19 and ULBP2/aCD19/aCD33 TriKEs) have demonstrated a meaningful *in vitro* and *in vivo* anti-tumor activity against CLL (71). Additionally, preclinical studies revealed that a CD16/CD19 BiKE and a CD16/CD19/CD22 TriKE are capable to trigger NK-cell activation through direct CD16 signaling (72). Recently, a potentiated CD16/CD19 TriKE structure (161519 TriKE), able to provide NK-cell expansion signal *via* an interleukin-15 moiety, was tested in both healthy donors- and CLL patients-derived samples. 161519 TriKE induced a potent activation of NK cells from healthy donors and a recovery of the cytotoxic functions of NK cells from CLL patients, resulting in enhanced NK-cell expansion and CLL target killing. Interestingly, 161519 TriKE also demonstrated to induce a better killing of CLL cells *in vitro* when compared with rituximab (73).

Taken together, these preliminary findings indicate that triggering an endogenous NK-cell response is compelling and support further investigations of BiKEs and TrIKEs as a therapeutic option for CLL patients.

### 3.2 Adoptive Cellular Therapy

#### 3.2.1 CAR T Cells: A Strategy to Combine mAb and T-Cell Mediated Cytotoxic Effects Against Leukemia

Amongst adoptive cellular therapies, CAR T cell-based treatment represents one of the most investigated fields of research, as significant results have been reported in B-cell haematological malignancies; more specifically, anti-CD19 CAR T cells are currently approved for clinical use in patients affected either by aggressive B-cell NHL, mantle-cell lymphoma or B-cell acute lymphoblastic leukemia.

CAR T cells combine components and features of both T cells and antibodies, thus remarkably enhancing T-cell anti-tumor activity. According to the composition and level of development, CARs constructs are divided in four generations (74). Currently, only 2^nd^ generation CAR constructs have been approved for clinical use, and these constructs consist of i) an antigen binding domain, which includes the single chain variable fragment (scFv) derived from an immunoglobulin directed against a tumor associated antigen, ii) an intracellular domain from the CD3 ζ- chain and iii) a costimulatory domain, generally identified as the intracellular signaling domains of a costimulatory molecule (i.e. CD28 or 41BB). The presence of the costimulatory domain allows the CAR-mediated full activation of T cells in the absence of interactions with antigen-presenting cells (74). The main advantage of CARs is their ability to recognize tumor antigens in an HLA-independent manner, thus being able to trigger immune responses even in an immune-evading tumor microenvironment (75).

Despite the remarkable efficacy obtained in other B-cell malignancies, CAR T-cell therapy use in CLL remains controversial. Previous studies of anti-CD19 CAR T cells in CLL showed an ORR between 50% and 70%, and only 20% to 30% of complete remission (CR) rates (76, 77). Nevertheless, updated data of the Transcend CLL04 study displayed an ORR of 82% with 45% of CR for CD19-targeting CAR T cells as single agent, resulting in a high rate of undetectable minimal residual disease (uMRD) in heavily pre-treated, high-risk CLL patients, including those refractory to both BTK inhibitors and venetoclax (78). Regardless of these encouraging results, CAR T-cell treatment is still a challenge in CLL. For example, the onset of resistance to CD19-directed CAR T cells due to CD19 antigen loss is not unusual, representing one of the most common causes of relapse (79). In order to optimize CAR T-cell products and to improve strategies to overcome the possible onset of resistance to therapy, today a variety of new constructs are being studied in numerous early-phase trials in CLL setting, including CARs that target alternative antigens, other than CD19 (e.g., CD20, CD22, ROR-1, Siglec-6), or that simultaneously target more than one antigen (e.g., CD19 and CD20, CD19 and CD22). In this context, a phase 1 trial exploring dual targeted anti-CD19/CD20 CAR T cells in relapsed or refractory B-NHL displayed limited toxicity and promising ORR, thus encouraging further investigation (80). With the aim of limiting B-cell aplasia and hypogammaglobulinemia that often occur with anti-CD19 CAR T cell-therapy (81, 82), a new CAR that selectively targets the immunoglobulin (Ig) light chain, which is restrictively expressed by the neoplastic clone but not by the normal B-cell compartment, is being explored. In this view, preclinical studies exploring the use of CAR T cells directed against Ig kappa or Ig lambda have been performed, reporting good activity both in *in vivo* and *in vitro* CLL models (83), and a clinical trial investigating anti-Ig kappa CAR T cells is currently ongoing (NCT04223765).

The main limitation of a successful use of CAR T cells in CLL is the occurrence of intrinsic dysfunctions of the T-cell compartment, that can interfere with the expansion and functionality of engineered T cells. In a study by Fraietta et al., it was demonstrated that CAR T cells from responder patients upregulated memory-related genes, with the enhancement of programs involved in cytokine production and in competent immune responses; differently, CAR T cells from non-responder patients were enriched in pathways involved in effector differentiation, glycolysis, exhaustion, and apoptosis (84). One possible strategy to overcome this limitation of CAR T-cell therapy in CLL is the co-administration of new targeted agents, able to partially restore the tumor microenvironment. It has been reported that treatment with at least 5 cycles of ibrutinib prior to CAR T-cell infusion can re-establish the normal T‐cell function and contribute to a better *ex vivo* expansion of CAR T cells with potentially enhanced *in vivo* functionality (85). More recently, Fan et al. demonstrated that the presence of ibrutinib during the manufacturing process produces an increase in the viability and expansion of patients‐derived CAR T cells, also restoring their underlying functional impairment (86). Additionally, ibrutinib demonstrated to improve the curative effects of CD19-directed CAR T cells in Raji cell subcutaneous tumorigenic mice, possibly thanks to its beneficial impact on the tumor microenvironment (87). In line with preclinical data, results from a phase 1 clinical trial confirmed the safety and feasibility of ibrutinib administered in combination with anti-CD19 CAR T cells in pretreated CLL patients (88). Interestingly, the administration of ibrutinib in combination with CAR T cells also resulted in an improved tolerability, as shown by the lower incidence of severe side effects, such as cytokine release syndrome or neurological events (88, 89). Similarly to ibrutinib, the novel BTK inhibitor acalabrutinib showed to improve the *in vitro* and *in vivo* anti-tumor functions of CD19-directed CAR T cells (90).

A new future perspective in the CAR T-cell therapy scenario is represented by the possibility of generating allogeneic T cells from a universal donor (uCAR T), with the twofold goal of overcoming the intrinsic functional limitations of patient-derived CAR T cells and of broadening the potential use of CAR T-cell treatment. Preclinical studies have already shown that CAR T cells generated from healthy donors perform more effectively than patient-derived CAR T cells, especially when compared to CAR T cells generated from patients with a high number of circulating leukemic cells or from patients refractory to autologous CAR T cells (84, 91). Up to date, it has been reported that the administration of allogeneic anti-CD19 CAR T cells resulted to be feasible, effective and safe in CLL patients who relapsed after HSCT (92–94). In this view, donors could be screened for a T-cell phenotype associated with a more efficient anti-leukemia effect, thus providing a standardised high-quality transfusion product. By contrast, transferring an allogenic CAR might induce GvHD to patients, or the allogeneic product might be rejected. With the aim of making uCAR T cells a safe and manageable therapy, genetic modification to remove the TCR (to limit GVHD) and/or HLA molecules (to limit rejection) are today under investigation (95).

#### 3.2.2 NK Cells: The Future of Adoptive Cellular Therapy?

NK cells may represent a valid alternative to T cells in the context of genetically-modified adoptive immunotherapy, mainly thanks to their ability to induce tumor cell killing in a MHC-unrestricted manner and without the need of prior exposure for activation (96). As a further advantage, NK cells can also be activated by natural cytotoxicity receptors and can eliminate tumor cells through a CD16-mediated ADCC, thus adding an additional death mechanism to the CAR-mediated cell lysis (97). Even more importantly, allogeneic NK cells can be safely administered without the need for full HLA-matching, thus avoiding the need of a custom-made production of one CAR-modified cellular product for each individual patient (98). As a further advantage, it has been shown that NK cells, while still retaining the ability to exert alloreactivity, do not to induce the onset of GvHD (99). For all the above reasons, NK cells are under extensive investigation for adoptive immunotherapy, even as genetically unmodified cellular products (100).

NK cells can be derived from multiple platforms, including peripheral blood, umbilical cord blood, and from NK92 cell lines, thus representing a valid “off-the-shelf” treatment (101). At the moment, cord blood-derived CAR NK cells seem to be the better option, thanks to their easy worldwide availability, their strong proliferation potential and the possibility to be manipulated during their manufacturing process in order to enhance their activation profile (101). Based on encouraging preclinical data, displaying a good activity of cord blood-derived CAR NK cells towards CLL cells (102), nowadays a few clinical trials are evaluating the feasibility of CAR NK-cell therapy in CLL (NCT01619761, NCT03056339). Preliminary evidences of the NCT03056339 trial established that allogeneic cord-blood derived CD19-directed CAR NK cells can be safely administered to relapsed or refractory CLL patients (103). Most importantly, results from this study suggest that CAR NK cell-therapy do not produce any major CAR T-cell treatment-related toxic effects, such as cytokine release syndrome or neurotoxicity, and, as expected, there was no evidence of GvHD (103). Unfortunately, in this study, the *in vivo* persistence of CAR NK cells, which today represents the main limitation for this kind of CAR-modified cellular therapy (104), could not be evaluated as treating physicians were allowed to perform post remission therapies after the 30 days-long assessment period.

In conclusion, based on currently available data, NK cell-based immunotherapy has encouraging therapeutic potential for the treatment of patients with solid and hematologic cancers, including CLL.

## 4 Combination Regimens as a Winning Strategy: The Example of the Use of Immune Checkpoint Inhibitors in CLL Transformed to Richter Syndrome

In recent years, ICI revolutionized cancer therapy thanks to their capability of eliciting anti-tumor responses by blocking inhibitory receptors or their ligands on immune cells. For instance, interactions of PD1 with its ligand PD-L1 represent a major immune checkpoint engaged by tumor cells to avoid T-cell immune surveillance. From the biological standpoint, little is known about PD1/PD-L1 axis in RS, however studies show how, differently from CLL, tumor cells in RS overexpress PD1 thus determining an impairment of T lymphocytes anti-tumor functions; for such reasons the PD1/PD-L1 axis could be a valid candidate for immunotherapy of RS (105). In CLL, PD1 inhibitors used as single agents have so far provided disappointing therapeutic results (60, 106), whereas the anti-PD1 mAb pembrolizumab has exhibited selective efficacy in patients with CLL transformed into RS (17, 107). Recently, clinical trials testing different approaches for RS showed a good activity of ICI when used in combination with novel agents. In this context, early results from trials combining ibrutinib with the anti-PD1 mAb nivolumab showed considerable results with acceptable toxicities (108, 109), and the triplet combination of umbralisib, ublituximab and pembrolizumab reported durable and sustained responses (110). In line with these results, the association between the Bcl-2 inhibitor venetoclax, the next-generation anti-CD20 mAb obinutuzumab and the anti-PD-L1 mAb atezolizumab led to high rates of response in previously untreated RS patients (111, 112), demonstrating one more time how immunotherapeutic approaches aiming a different targets can exploit a synergistic action against tumor cells. Currently, several ongoing clinical trials are evaluating the combination of anti-PD1 immune checkpoint inhibitor and ibrutinib (phase I/Ib NCT04781855 and phase II NCT02420912), or the PI3K inhibitors copanlisib (phase I NCT03884998) and duvelisib (phase I NCT03892944).

## 5 Conclusions

Over the time several immune-based therapeutic options have been explored in the CLL context, with the ultimate goal of exploiting and/or boosting the patient’s immune system to fight tumor cells. Despite encouraging preclinical data showing significant anti-tumor activity, immune-based therapies often achieved suboptimal results in terms of tumor control, possibly due to intrinsic immune defects hampering the obtainment of a powerful therapeutic response. A demonstration of these limitations is provided by the disappointing results obtained with active immunotherapy strategies used individually, which have shown to be ineffective in re-directing against the tumor an immune system that was not competent enough. Conversely, the use of agents that display immunomodulatory properties reported successful results, especially in combination settings.

Interestingly, targeted agents currently used in the treatment of patients with CLL (i.e., BTK inhibitors, PI3K inhibitors, and the Bcl-2 inhibitor venetoclax) have shown not only to act against malignant B cells but also to exert at some degree an immunomodulatory activity through mechanisms that are not necessarily connected to their on-target effects. For example, ibrutinib, although designed to inhibit BTK downstream the B-cell receptor, is also able to exert off-target effects on the T-cell compartment – such as an increase in the T-cell number and function (113) and a TH1 polarization (114) - through the binding of the IL-2-inducibile T cell kinase (ITK). Consistently, long-term treatment with ibrutinib has also demonstrated to reduce the expression of exhaustion markers and inhibitory checkpoints on T cells (115, 116). The ability of re-converting the immune system from CLL patients to a more effective status has led to the addition of ibrutinib to other regimens, with significant improvement of tumor control. Preclinical and clinical results have already demonstrated the ability of ibrutinib in i) improving the efficacy of the novel mAbs cirmtuzumab (52, 53), ianalumab (59) and BI 836826 (48), ii) enhancing the susceptibility of CLL cells to BiTE-mediated killing (66, 67), and iii) potentiating the generation, functionality and curative effects of CAR T cells (88, 89). Similarly, other agents capable of modulating the immune system, such as avadomide, have shown promising results when administered in combination with checkpoint inhibitors (23), thus leading to reconsider active immunotherapy as a potential therapeutic option for patients with CLL.

Based on these evidences, one promising approach currently under investigation is the design of combination regimens exploiting the potential synergism of novel agents and immunotherapy, with the aim of achieving a deeper and more persistent eradication of leukemic cells, and therefore a potential cure of CLL.

## Author Contributions

FP and RJ reviewed the literature and wrote the manuscript. VG contributed to literature review and to manuscript revision. CV and MC designed the review and revised the manuscript. CV and MC equally contributed to this work. All authors participated in the writing, read, and approved the submitted version.

## Funding

This research received no external funding. VG was a recipient of a fellowship from the “Associazione Damiano per l’Ematologia”.

## Conflict of Interest

CV has received consultancy fees and honoraria from Janssen. MC received honoraria from Janssen, Gilead, Abbvie, Shire, Astrazeneca and research support from Janssen, Abbvie and Karyopharm Therapeutics.

The remaining authors declare that the research was conducted in the absence of any commercial or financial relationships that could be construed as a potential conflict of interest.

## Publisher’s Note

All claims expressed in this article are solely those of the authors and do not necessarily represent those of their affiliated organizations, or those of the publisher, the editors and the reviewers. Any product that may be evaluated in this article, or claim that may be made by its manufacturer, is not guaranteed or endorsed by the publisher.
